# Purification and characterization of tyrosinase from walnut leaves (*Juglans regia*)

**DOI:** 10.1016/j.phytochem.2014.02.010

**Published:** 2014-05

**Authors:** Florime Zekiri, Christian Molitor, Stephan G. Mauracher, Claudia Michael, Rupert L. Mayer, Christopher Gerner, Annette Rompel

**Affiliations:** a Institut für Biophysikalische Chemie, Universität Wien, Althanstraße 14, A-1090 Wien, Austria; b Institut für Analytische Chemie, Universität Wien, Währinger Straße 38, A-1090 Wien, Austria

**Keywords:** *Juglans regia*, Type-3 copper protein, Polyphenol oxidase, Tyrosinase, Catechol oxidase, Laccase, ACN, acetonitrile, CT, charge transfer, ESI-MS, Electrospray Ionisation Mass Spectrometry, FPLC, Fast Protein Liquid Chromatography, IEF, isoelectric focusing, *Jr*, *Juglans regia*, nanoESI-QTOF, nanoElectrospray ionisation quadrupole-time-of-flight mass spectrometry, LC–MS/MS, liquid chromatography–mass spectrometry/mass spectrometry, MS, mass spectrometry, nanoUHPLC–ESI-MS/MS, nanoUltra high performance liquid chromatography–electrospray tandem mass spectrometry, PEG-4000, polyethylene glycol 4000, PMSF, phenylmethylsulfonyl fluoride, PPO, polyphenol oxidase, SDS, sodium dodecyl sulfate, SDS–PAGE, sodium dodecyl sulfate–polyacrylamid gel electrophoresis, UV/Vis spectroscopy, ultraviolet/visible spectroscopy

## Abstract

Polyphenol oxidase (PPO) is a type-3 copper enzyme catalyzing the oxidation of phenolic compounds to their quinone derivates, which are further converted to melanin, a ubiquitous pigment in living organisms. In this study a plant originated tyrosinase was isolated from walnut leaves (*Juglans regia*) and biochemically characterized. It was possible to isolate and purify the enzyme by means of an aqueous two-phase extraction method followed by chromatographic purification and identification. Interestingly, the enzyme showed a rather high monophenolase activity considering that the main part of plant PPOs with some exceptions solely possess diphenolase activity. The average molecular mass of 39,047 Da (Asp^101^ → Arg^445^) was determined very accurately by high resolution mass spectrometry. This proteolytically activated tyrosinase species was identified as a polyphenol oxidase corresponding to the known *jrPPO1* sequence by peptide sequencing applying nanoUHPLC–ESI-MS/MS. The polypeptide backbone with sequence coverage of 96% was determined to start from Asp^101^ and not to exceed Arg^445^.

## Highlights

• A tyrosinase from walnut leaves (*Juglans regia*) was purified.
• The *Juglans regia* enzyme was determined to have a mass of 39,047 Da and a p*I* of 5.2.
• Addition of 2 equivalent H_2_O_2_ leads to *oxy*tyrosinase.
• The proteolytically activated tyrosinase was clearly identified as PPO1 *Juglans regia*.

## Graphical abstract

Tyrosinase from walnut leaves (*Juglans regia*) corresponding to the known *jrPPO1* sequence was purified and characterized. Two major tyrosinase forms differing only in their C-termini were identified. The first form (*jrPPO1*(Asp^101^ → Pro^444^)) is one amino acid shorter than the second form (*jrPPO1*(Asp^101^ → Arg^445^)).

**Figure:**
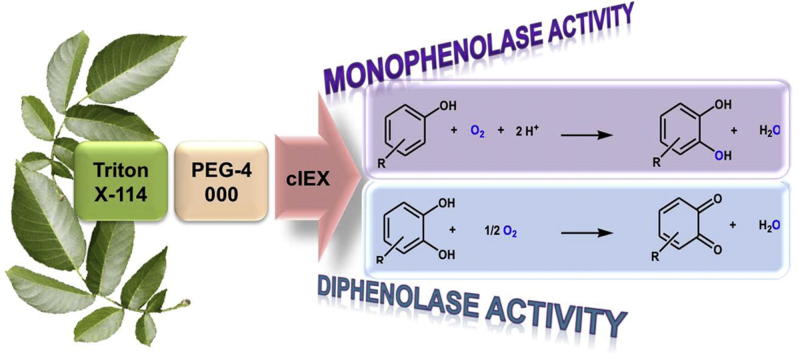

## Introduction

Polyphenol oxidases (PPO) are metalloenzymes containing a type-3 copper center occurring in many organisms including plants, fungi and bacteria (Mayer, 2006; Selinheimo et al., 2007). Representatives of this class are catechol oxidases, tyrosinases and laccases. Tyrosinases, a class of bifunctional PPOs, use molecular oxygen to catalyze the oxidation of various monophenols to *o*-diphenols (cresolase/monophenolase activity; EC 1.14.18.1), and the subsequent oxidation of *o*-diphenols to the corresponding *o*-quinones (catecholase/diphenolase activity; EC 1.10.3.1). Catechol oxidase catalyzes exclusively the oxidation of *o*-diphenols to *o*-quinones, lacking the hydroxylation reaction (Mayer, 2006; Mayer and Harel, 1979). Laccases (EC 1.10.3.2) can oxidize a wide range of compounds including aminophenols, monophenols, *o*- and *p*-diphenols by removing single electrons from the reducing group of the substrate and generate free radicals (Mayer and Harel, 1979; Sanchez-Amat and Solano, 1997).

Melanines generated by polymerisation of quinones are dying-compounds which are responsible for the damage-induced browning of many fruits and vegetables (Martinez and Whitaker, 1995; Yoruk and Marshall, 2003). While the physiological function of PPOs in many plants is still not clear, there is strong evidence that PPOs play a key role in parasite and pathogen resistance in some species. Protective effects of PPOs have generally been attributed to the generation of reactive quinones (Steffens et al., 1994; Van Gelder et al., 1997; Vaughn et al., 1988).

Type-3 copper proteins contain two copper ions, each coordinated by three histidine residues. During the catalytic reaction, the type-3 copper center of tyrosinase exists in three different states. The reduced *deoxy* state [Cu(I)–Cu(I)] binds molecular oxygen and results in the *oxy* state [Cu(II)–O_2_^2−^–Cu(II)]. In the *oxy* state, peroxide is bound in a μ–η^2^:η^2^ bridging mode (Kitajima et al., 1989). The *met* state [Cu(II)–Cu(II)] is assumed as the resting state of the copper site, where Cu(II) ions are bridged by a water molecule or hydroxyl ion (Solomon et al., 1996).

The functional importance of the PPO-activity in walnut hull was first described in 1991 (Piffaut and Metche, 1991). Walnut (*Juglans regia*) PPO was attributed to possess a putative pathogenic resistance as early as 1911 (Cook et al., 1911). Walnut leaves have a high content of various polyphenols, some of which might be important in pathogenic resistance (Colaric et al., 2005; Solar et al., 2006). PPO from walnut leaves has been poorly studied in the past (Escobar et al., 2008; Piffaut and Metche, 1991). Escobar et al. (2008) demonstrated that PPO of walnut is encoded by a single gene, *jrPPO1*, which is constitutively expressed in all green, herbaceous tissues of walnut.

In this work a tyrosinase from walnut leaves (*J.* *regia)* is extracted and purified by means of fast protein liquid chromatography (FPLC) and characterized by molecular mass determination (SDS–PAGE, nanoESI-QTOF), isoelectrical focusing (IEF), UV/Vis spectroscopy and sequence analysis (nanoUHPLC–ESI-MS/MS). The purification method described in this paper allows a very efficient isolation of two forms from walnut tyrosinase, identified and characterized by mass spectrometry based methodology providing sequence information and the highly accurate mass.

## Results and discussion

### Extraction and purification of tyrosinase from *J. regia*

The applied extraction method was based on a technique described for a latent tyrosinase from mushrooms (*Agaricus bisporus*) and had proven to be effective (Mauracher et al., 2014). Several consecutive aqueous two-phase separations using triton X-114 and PEG-4000 (polyethylene glycol) resulted in a quantitative removal of hydrophobic dyes, non-target proteins and other hydrophobic compounds. Hence, the obtained polyphenol free and clear protein solution was very well suitable for the subsequent chromatographic purification steps.

**Fig. 1:**
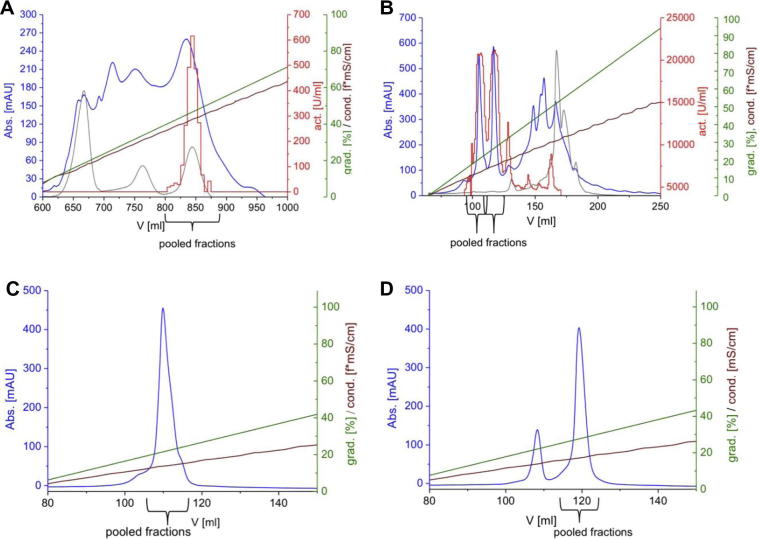
Chromatographic runs (FPLC). (A) Cation exchange chromatography on SP-Sepharose. (B) Cation exchange chromatography on MonoS. (C) Cation exchange chromatography on MonoS *jrPPO1*(Asp^101^ → Pro^444^). (D) Cation exchange chromatography on MonoS *jrPPO1*(Asp^101^ → Arg^445^). Legend: , UV absorbance at 280 nm [mAU]; , UV absorbance at 410 nm [mAU]; , monophenolase activity [U/ml]; , gradient [% buffer B]; , conductivity [mS/cm] (f = ∼1.5).

Using a cation exchange column (SP-Sepharose) as a first purification step proved being very effective in terms of removing a major part of non-target protein. The target PPO eluted late in the sodium chloride gradient together with one of in total three co-eluted heme-proteins. This is clearly shown in Fig. 1A by following the absorption at 410 nm characteristic for prosthetic heme groups. Implying some characterization experiments (data not shown) it can be assumed that the interfering protein is a peroxidase. This was also observed by Trémolières and Bieth (1984), Rompel et al. (1999a, 2012). Fractions showing highest tyrosinase activity were pooled and loaded onto the second cation exchange column (MonoS) where two major forms of the tyrosinase, which are named after the chromatographic elution order, forms 1 and 2, were eluted consecutively but efficiently separated early in the gradient (see Fig. 1B). On the cation exchange column (MonoS) the remaining heme protein (peroxidase) could be very effectively separated from the tyrosinase (see Fig. 1B). For polishing reasons fractions of the two tyrosinase forms were separately applied to the same cation exchange column (MonoS) resulting in a very high purity of the two tyrosinase species (see Figs. 1C/D and 2).

Often isoforms of polyphenol oxidase were found during isolation and purification. Currently six amino acid sequences (PPO1 to 6) are known for a polyphenol oxidase originating from *Agaricus bisporus* (Li et al., 2011*;* Mauracher et al., 2014; Weijn et al., 2013; Wichers et al., 2003; Wu et al., 2010). Eicken et al. (1998) isolated two catechol oxidases with different amino acid sequences from sweet potato (*Ipomoea batatas)* having molecular masses of 39 and 40 kDa, respectively. Four PPO isoforms were purified from coats and pods of green bean (*Phaseolus vulgaris L.*), and their molecular weights were estimated to be 57.5, 54, 46 and 39 kDa, respectively (Guo et al., 2009). In this manuscript it is demonstrated that two forms of walnut tyrosinase, arising from the same gene (*jrPPO1*, Escobar et al., 2008), but proteolytic cleaved on different positions were purified and characterized.

### Electrophoresis study

**Fig. 2:**
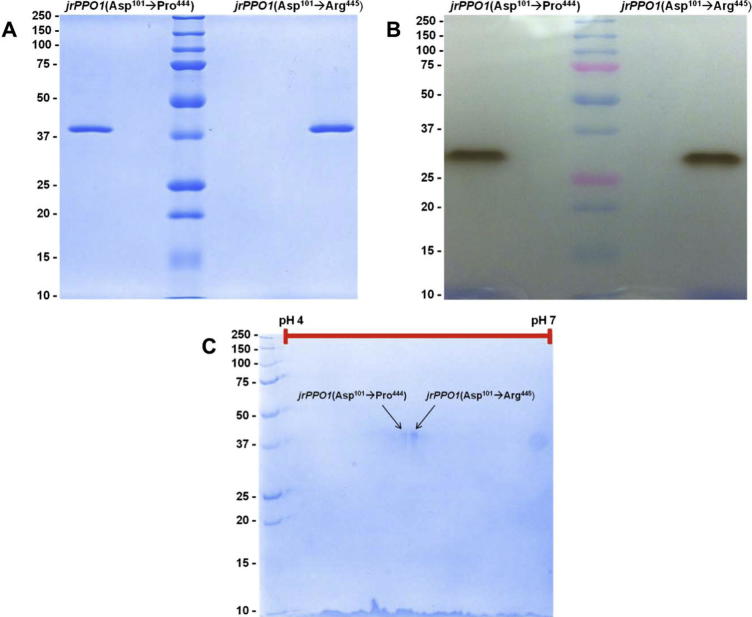
Analytic one- and two-dimensional SDS–PAGEs. (A) Purified *jrPPO1*(Asp^101^ → Pro^444^) and *jrPPO1*(Asp^101^ → Arg^445^). Staining: Coomassie Brilliant Blue. M_w_ marker [kDa] in the middle lane. (B) In-gel activity staining by l-tyrosine. M_w_ marker [kDa] in the middle lane. (C) Coomassie-stained two-dimensional polyacrylamide gel of *jrPPO1*(Asp^101^ → Pro^444^) and *jrPPO1*(Asp^101^ → Arg^445^), which were separated in the first dimension on an immobilized pH gradient strip (pH 4.0–7.0) and in the second dimension on a 12% polyacrylamide gel.

Purity of the two tyrosinase forms was determined by sodium dodecyl sulfate polyacrylamide gel electrophoresis (SDS–PAGE). Whereas no bands corresponding to a non-target protein were visible, both forms showed a single band at around 39 kDa (see Fig. 2A). The in-gel activity was measured following non reducing SDS–PAGE as the reduction is detrimental to the activity of the enzyme. In this non reduced form the enzyme displays a higher electrophoretic mobility, which is probably due to its more compact conformation. Under non-reducing conditions the intramolecular disulfide bridges are intact and can therefore stabilize the enzyme’s conformation (see Fig. 2B). Moreover, the IEF (both samples applied) resulted in two spots at a p*I* of 5.1 and 5.2, respectively (see Fig. 2C). This matches with the theoretically calculated p*I* of 5.25 (*ProtParam,* ExPASy.org) corresponding to the identified amino acid sequence *jrPPO1*(Asp^101^–Pro^444^) and p*I* of 5.35 for *jrPPO1*(Asp^101^–Arg^445^) (UniProt.: COLU17) as described below.

### Protein identification and sequence confirmation

**Fig. 3:**
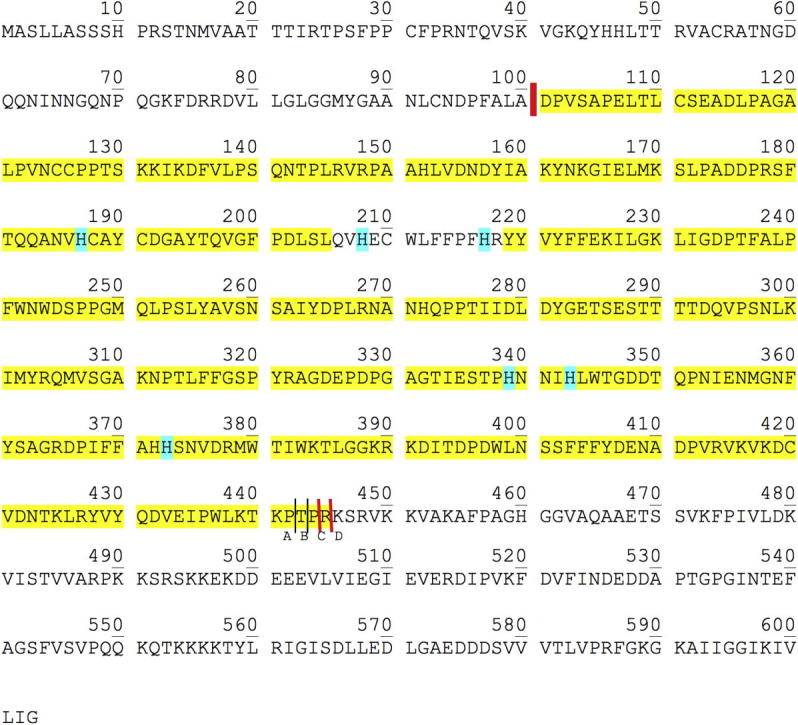
Sequence of *jrPPO1.* Highlighted are: , copper coordinating histidines; , Peptides identified by nanoUHPLC–ESI-MS/MS are highlighted yellow. Red lines () indicates the start/end of the *jrPPO1*(Asp^101^ → Pro^444^) and *jrPPO1*(Asp^101^ → Arg^445^) sequence, respectively, as deduced from matching with the molecular mass determined for the isolated protein by nanoESI-QTOF. Black thin lines () indicate the cleavage positions of the minor sub-species (A, B) only found in *jrPPO1*(Asp^101^ → Pro^444^).

The isolated and purified two forms of tyrosinases were clearly identified as PPO1 *J.* *regia* (UniProt.: COLU17, Escobar et al., 2008) by means of nanoUHPLC–ESI-MS/MS yielding a maximum sequence coverage of 96% (*jrPPO1*(Asp^101^ → Pro^444^)) and 96% (*jrPPO1*(Asp^101^ → Arg^445^)) yellow highlighted in Fig. 3. 91 (*jrPPO1*(Asp^101^ → Pro^444^)) and 71 (*jrPPO1*(Asp^101^ → Arg^445^)) tryptic peptides were identified and listed in Table 1 (*jrPPO1*(Asp^101^ → Pro^444^)) and Table 2 (*jrPPO1*(Asp^101^ → Arg^445^)). Hence, the two separated and purified tyrosinase forms distinguish themselves in possessing or lacking the terminal amino acid Arg^445^. This was confirmed as well by the mass determination of the intact protein by nanoESI-QTOF.

Whereas, an almost complete peptide coverage of the main core region was found the preceding transit peptide region (Met^1^–Ala^100^) as well as the *C*-terminal part (Lys^446^–Gly^603^) are missing (Flurkey and Inlow, 2008). Giving the fact that protease inhibition agents (PMSF, benzamidine hydrochloride, both serine-protease inhibitors) were used during extraction, it can be assumed that proteolytic removal of the enzymes’ *C*-terminal part happens *in vivo* or is caused by non-serine proteases affection. The peptide carrying the common thioether bridge (Gielens et al., 1997; Klabunde et al., 1998) found for all known eukaryotic PPOs in literature could not be detected. Cysteine–histidine thioether bridges are reported for the sequences of *I.* *batatas* catechol oxidase (Klabunde et al., 1998), *Neurospora crassa* tyrosinase (Lerch, 1982), *Vitis vinifera* polyphenol oxidase (Virador et al., 2010) and mushroom tyrosinases (Ismaya et al., 2011; Mauracher et al., 2014; Van Gelder et al., 1997).

### Molecular mass determination

**Fig. 4:**
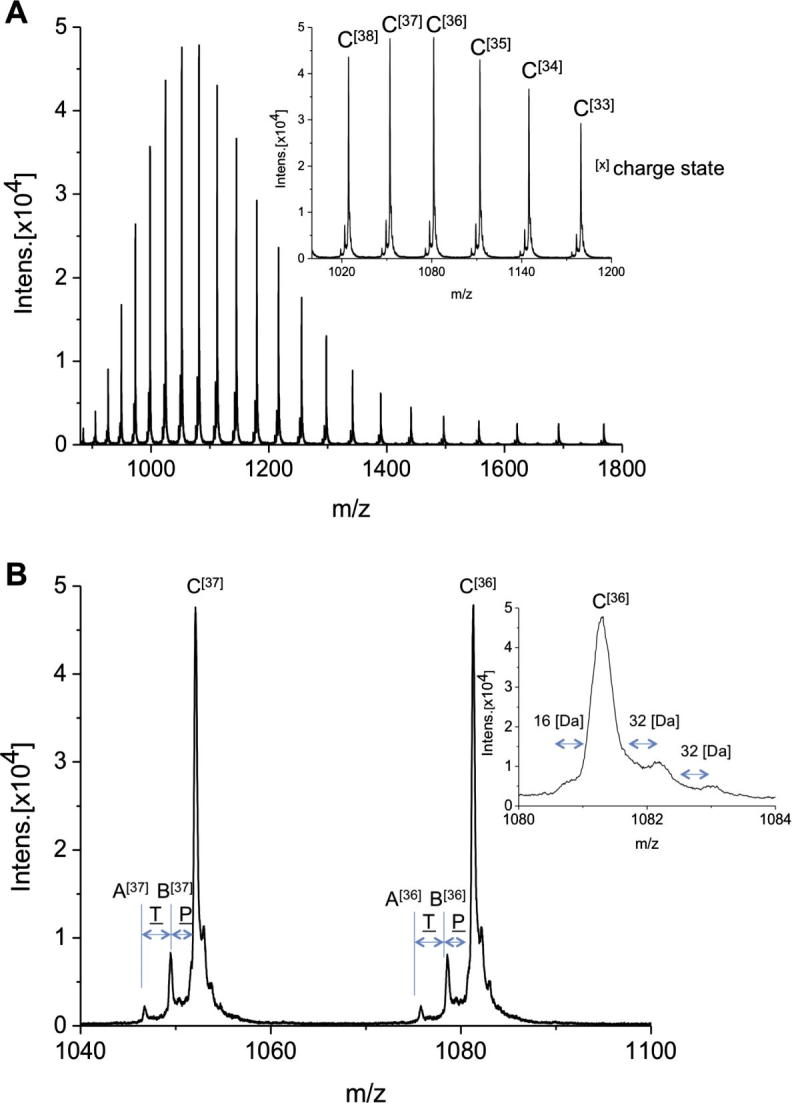
(A) nanoESI-QTOF mass spectra of *jrPPO1*(Asp^101^ → Pro^444^). Inset^(A)^: Peaks of charge state [33] to [38] magnified. (B) Zoomed section of charge state [36] and [37]. Masses of species A–C: A = 38,692 Da, B = 38,793 Da; C = 38,890 Da; Mass differences between species fit to distinct amino acid composition indicated in amino acid letter code. Inset^(B)^: Highly zoomed figure of charge state [36] showing clearly shoulders corresponding to mass differences of 16 or 32 Da (oxidation).

The mass spectrum in Fig. 4 shows the first species *jrPPO1*(Asp^101^ → Pro^444^) obtained by the nanoESI-QTOF instrument with resolution (FWHM) of 40,000 and a mass accuracy of better than 5 ppm. The charge state distributions shown in inset (a) of Fig. 4A (ranging from about 28 to up to more than 40 charges) indicates the presence of one major protein species C when assorting by increasing M_r_ and two minor abundant ones A, B. A zoomed-in section of this spectrum is shown in Fig. 4B. The magnified inset (b) shows unambiguously that each species peak has shoulders (Δm ≈ 16 Da or 32 Da) presumably due to the presence of oxidized and non-oxidized species. For the deconvolution of the charge state distribution shown in inset (a) (Fig. 4A), 15 distinct peaks were used for the major species C, and at least 10 peaks with sufficient signal to noise ratios for the species A and B. Assuming that these positive charge (z) states are solely caused by the attachment of z protons the average molecular masses for the major species C can be assessed as 38,890 Da (STD less than 0.1 Da) and A = 38,692 Da; B = 38,793 Da (STD less than 1 Da), respectively.

**Fig. 5:**
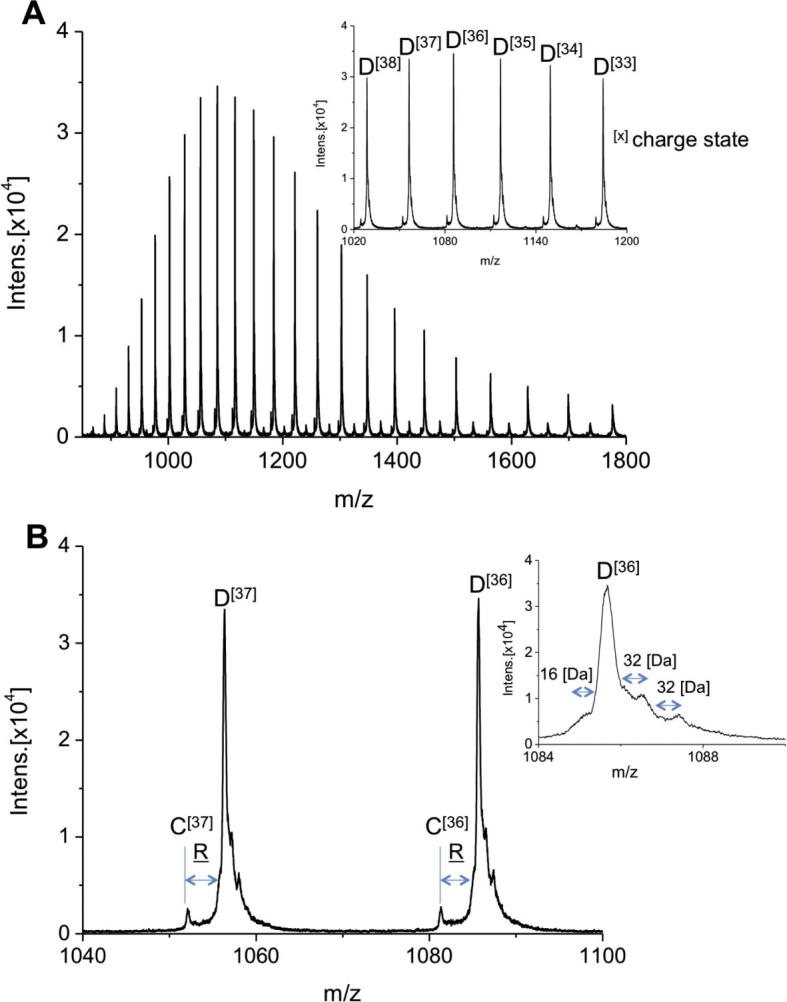
(A) nanoESI-QTOF mass spectra of *jrPPO1*(Asp^101^ → Arg^445^). Inset^(A)^: Peaks of charge state [33] to [38] magnified. (B) Zoomed section of charge state [36] and [37]. Masses of species C–D: C = 38,890 Da, D = 39,047 Da; Mass difference between species fit to distinct amino acid composition indicated in amino acid letter code. Inset^(B)^: Highly zoomed figure of charge state [36] showing clearly shoulders corresponding to mass differences of 16 or 32 Da (oxidation).

Fig. 5 shows the mass spectrum of *jrPPO1*(Asp^101^ → Arg^445^). The charge state distributions shown in inset (a) in Fig. 5A (ranging from about 28 to up to more than 40 charges) indicates the presence of one major protein species D, when assorting by increasing M_r_ and one minor protein species C. The average molecular masses for the major species D can be assessed as 39,047 Da (STD less than 0.1 Da) and C = 38,890 Da (STD less than 1 Da).

The nanoESI-QTOF-MS measurements gave evidence for the presence of two major species C (Asp^101^ → Pro^444^), D (Asp^101^ → Arg^445^) and two minor species A, B (occur together with C) that are reasonably deduced as protein forms being proteolytically cleaved at different positions of the main core polypeptide-chain-end as specified in Figs. 4B and 5B. The mass differences between these proteolytic species fit accurately to the amino acids Pro^442^, Thr^443^, Pro^444^, and Arg^445^ (see Fig. 3). This is additionally confirmed by the peptide mass analyses which found all kinds of possible main core end peptides (see Fig. 3). Thus, it can be assumed that either the proteolytic removal of the enzymes’ *C*-terminal part has no distinct preferential cleavage site but a region (Pro^442^–Arg^445^), or the distinct site is Arg^445^, however, *C*-terminal fringing is caused by *C*-terminal exo-peptidases *in vivo* or during extraction.

### Kinetic parameters

Parameters obtained by kinetic Michaelis–Menten measurements are reported in Table 3. Giving a *k*_cat_ value of 20.8 s^−^^1^ toward l-tyrosine and a *k*_cat_ value of 199.3 s^−^^1^ toward l-dopa the enzyme possess a higher monophenolase- and diphenolase activity as enzymes in literature e.g. *Agaricus bisporus* tyrosinase with *a k*_cat_ value of 7.9 s^−1^ (l-tyrosine) and 107.4 s^−1^ (l-dopa) (Espín et al., 2000) and *Bacillus megaterium* tyrosinase with a *k*_cat_ value of 4.0 s^−1^ (l-tyrosine) and 44.1 s^−1^ (l-dopa) (Goldfeder et al., 2013). Notably, whereas tyrosinases from fungi, bacteria and mammals are kinetically well characterized, only catechol oxidases from plants received comparable attention in literature (Eicken et al., 1998; Rompel et al., 1999b). The relative high *k*_cat_ values of the purified *jrPPO1* results in a classification as a tyrosinase.

### UV/Vis spectroscopic studies

**Fig. 6:**
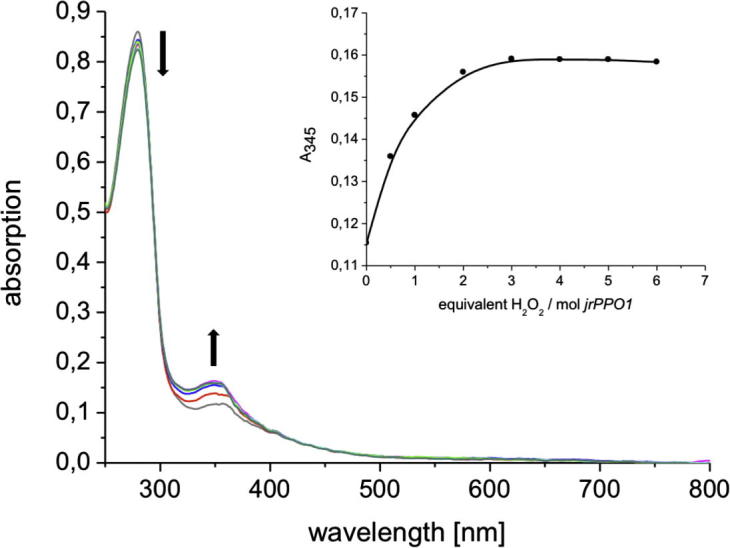
UV/Vis spectra of the native tyrosinase in 0.05 M HEPES pH 7 after treatment with 0 equivalents, 0.5, 1, 2, 3, 4, 5, and 6 eq. H_2_O_2_; T = 25 °C – Inset: absorption at 345 nm vs. equivalents H_2_O_2_.

The UV/Vis spectrum of the native *jrPPO1*(Asp^101^ → Pro^444^) is presented in Fig. 6. The absorption maximum occurs at 280 nm and a significant shoulder at 292 nm indicates the presence of several tryptophan residues in the amino acid sequence. 16 tyrosines, 18 phenylalanines, 8 tryptophans and 9 histidines are present in the mature *jrPPO1*(Asp^101^ → Pro^444^), which are also found in similar amounts in the catechol oxidase amino acid sequence from *I.* *batatas* (Eicken et al., 1998; Klabunde et al., 1998). For all type-3 copper enzymes a weak absorption maximum at 345 nm is observed, due to the charge transfer (CT) transition O_2_^2−^ (π^∗^_σ_) → Cu (II) (d_x^2^_ _−_ _y^2^_). Addition of H_2_O_2_ causes an increasing absorption band at 345 nm (*ε*_345_ = 12,984 M^−1^ cm^−1^ per protein) and 580 nm (*ε*_580_ = 761 M^−1^ cm^−1^ per protein). The absorption band at 580 nm corresponds to the second O_2_^2−^ (π^∗^_v_) → Cu (II) (d_x^2^_ _−_ _y^2^_) CT transition as found in *oxy* hemocyanin and *oxy* tyrosinase (Eickman et al., 1979; Himmelwright et al., 1980; Jolley et al., 1974; Solomon et al., 1992, 1994). The saturation of *jrPPO1* is reached with the addition of two equivalents H_2_O_2._ This result is very similar to the catechol oxidase from *Melissa officinalis* and to the 40 kDa catechol oxidase from *I.* *batatas* where addition of two equivalents of H_2_O_2_ led to saturation (Eicken et al., 1998; Rompel et al., 2012).

## Concluding remarks

By applying a modified and adjusted purification method published before from our group (Mauracher et al., 2014) two forms of walnut tyrosinase from the same gene (*jrPPO1*, Escobar et al., 2008), however proteolytically digested at different positions, were efficiently isolated and purified to identity. The accurate molecular mass was determined by means of high resolution mass spectrometry allowing the deduction of the enzymes polypeptide backbone. Thus, the *in silico* backbone masses of Asp^101^ → Pro^444^ and Asp^101^ → Arg^445^ matched with the determined molecular masses of 38,890 and 39,047 Da, respectively. This was also confirmed by mass spectrometric peptide analyses. The UV/Vis spectroscopic results verified that the tyrosinase from *J.* *regia* contains a type-3 copper center. After addition of two equivalents H_2_O_2_ the full *oxy* form of the tyrosinase is developed. Kinetic experiments classified the enzyme as a tyrosinase (EC. 1.14.18.1 and EC. 1.10.3.1) possessing comparable catalytic activity towards l-tyrosine as other well characterized tyrosinases.

## Experimental

### Plant material

Walnut leaves were harvested from several trees in the surroundings of Vienna (June–September 2012) and stored at −80 °C in a freezer until used.

### Extraction of tyrosinase from *J. regia*

Tyrosinase was extracted as described by Mauracher et al. (2014) with some modifications. About 1 kg of frozen leaves were mixed and suspended in 2 L extraction buffer (125 mM sodium citrate, 4% (v/v) triton X-114, 0.5% (w/v) sodium ascorbate, 40 mM l-proline, 2 mM benzamidine hydrochloride, 1 mM phenylmethylsulfonyl fluoride (PMSF). The suspension was centrifuged at 14,500 rpm (Beckmann XP26, rotor: JLA 16.250) for 15 min at 4 °C. The supernatant was filtered through a coarse filter (cheesecloth) and treated with ammonium sulfate (15 g/L) to obtain a phase separation. This process was supported by centrifugation (14,500 rpm at 15 °C for 15 min) and allowed removal of the detergent-rich phase (lower phase). The supernatant containing soluble tyrosinase was brought to 30% (176 g/L) saturation with ammonium sulfate under continuous stirring at 4 °C. After 30 min of stirring the solution was centrifuged (14,500 rpm) for 30 min at 4 °C and the pellet was discarded. For further purification, PEG-4000 was dissolved to a concentration of 4% (w/v) in the supernatant at 4 °C. Following a centrifugation (14,500 rpm, 4 °C, 10 min) the generated PEG phase (upper phase) was discarded. This step was followed by the subsequent addition of 3.5% (w/v) PEG-4000 and centrifugation (14,500 rpm at 15 °C for 15 min) which was repeated twice. The purified and clear supernatant containing the soluble tyrosinase was brought to 80% (351 g/L) saturation with ammonium sulfate and stored overnight at 4 °C. The obtained protein pellet was then filtered through a bottle top filter and resuspended in 20 mM sodium acetate buffer, pH 4.5. The solution was then centrifuged at 14,500 rpm (4 °C) for 30 min and the supernatant was diluted with 20 mM sodium acetate buffer, pH 4.5 until the conductivity was below 9 mS/cm.

### Purification of tyrosinase by Fast Protein Liquid Chromatography

The supernatant was loaded onto a cation exchange column (SP-Sepharose FF, *GE Healthcare*, length = 10 cm, diameter = 2.6 cm) and equilibrated with 20 mM sodium acetate, pH 4.5. Bound proteins were eluted with a linear gradient of sodium chloride (0–1 M) at a flow rate of 5 mL/min (see Fig. 1A). All collected fractions were tested photometrically for monophenolase and diphenolase activity. Fractions containing activity were pooled, ultra filtrated (size exclusion membrane of 10 kDa) and centrifuged (4000 rpm, 4 °C) to remove sodium chloride. The protein solution was then applied to a MonoS HR 5/50 Gl column (cation exchange column, *GE Healthcare,* length = 50 mm*,* diameter = 5 mm) and eluted with a linear gradient of sodium chloride (0–0.7 M) at a flow rate of 1 mL/min. Two forms were eluted at a conductivity of 13 and 16 mS/cm (see Fig. 1B), respectively. Fractions containing the first and the second eluted protein form were separately pooled and again loaded on the MonoS HR 5/50 Gl column under same conditions for removing further non-target proteins in a final polishing step (see Fig. 1C/D).

### Enzyme activity assay during purification

The activity of the enzyme during the purification was monitored by spectrophotometric measurements (*SHIMADZU UV-1800*). Two different enzyme assays were performed. Monophenolase activity was determined by measuring the rate of increase absorbance at 305 nm and 25 °C. The reaction was performed in a 1 cm quartz cuvette using 1 mL of 10 mM sodium phosphate buffer, pH 6.5 containing 2.5 mM SDS, 0.033 mM l-tyrosine as substrate and 10 μL of enzyme solution. Diphenolase activity was determined through absorbance at 400 nm and 25 °C. The reaction mixture (1 mL) consisted of 125 mM sodium citrate buffer, pH 5.4, 5 mM 4-tert-butylcatechol as substrate and 1 μL of enzyme solution. One unit (U) of enzyme activity is defined as a change in 1 absorbance unit/min/ml.

### Protein concentration

Protein concentration was determined by the method of Bradford (1976) using bovine serum albumin (BSA) as standard.

### Enzyme kinetic analysis

The Michaelis–Menten constant (*K*_m_) and maximum reaction velocity (*V*_max_) were determined using two substrates (l-dopa and l-tyrosine) at five different concentrations (1.96, 1.30, 0.98, 0.49 and 0.29 mM). The substrates were dissolved in 50 mM sodium phosphate buffer pH 6.5. Data were plotted as 1/*V* and 1/[S] concentration according to the method of Lineweaver and Burk (1934).

### Isoelectrical focusing

Isoelectrical focusing was performed using a PROTEAN IEF cell (*Bio-Rad*) and IPG-stripes with the pH range 4–7 as marker.

### Molecular mass determination

The molecular mass of the purified enzyme was determined by denaturating SDS–PAGE. SDS–PAGE was performed according to the method of Laemmli (1970) using Precision Plus Protein Standard Dual Color (*Bio-Rad*) as molecular weight marker. Samples were applied to 10% polyacrylamide gels mixed with reduced loading dye. Gels were stained with Coomassie Brilliant Blue. The target protein band was cut out and used for protein identification. For in-gel tyrosinase activity staining a partially denaturating 10% SDS–PAGE was performed as described above, but without ß-mercaptoethanol in the loading dye, to preserve the enzyme activity. The gel was soaked with 10 mM sodium phosphate buffer pH 6.5 containing 0.033 mM l-tyrosine for activity staining. Imaging of the gels was done with Gel Doc™ XR of *BIO-RAD*.

Electrospray Ionisation Mass Spectrometry (ESI-MS) was performed on a nanoESI-QTOF mass spectrometer (maxis 4G UHR-TOF, *Bruker*, coupled to a nanospray robotic device, *Nanomate, Advion Biosiences,* voltage: 1.4 kV, dry gas: 6.0 l/min, dry heater 150 °C). Prior to MS measurements, the purified enzyme solution was ultra filtrated by centrifugation (14,000 rpm) and the buffer system was changed to 5 mM ammonium acetate pH 5.0, in order to reduce the salt concentration to a minimum. Afterward acetonitrile (ACN) and formic acid were added to a final concentration of 25% (v/v) ACN and 0.05% (v/v) formic acid.

### UV/Vis spectroscopic studies

Visible and ultraviolet spectra were monitored by a 2-beam cuvette photometer (*SHIMADZU UV-1800*) with 10 mM sodium acetate buffer pH 5.0 as reference (220–800 nm). The samples were tested in a quartz cuvette with coat thickness of 1 cm path length. The peroxo complex was prepared by adding 0.50–6.00 eq. H_2_O_2_ to tyrosinase in a 50 mM HEPES buffer, pH 7.0.

### Protein identification and sequence confirmation

2 μg of both protein forms were further purified for LC–MS/MS analysis by 1D SDS–PAGE. The proteins were visualized by Coomassie staining and the bands corresponding to the forms excised. The gel slices were destained with 30% acetonitrile/100 mM NH_4_HCO_3_ followed by cysteine carbamidomethylation applying dithiothreitol and iodoacetamide. The digestion was carried out with trypsin as well as chymotrypsin and the peptides eluted from the gel slices by ultrasonication. The peptide samples were completely dried by vacuum centrifugation and stored at −20 °C for LC–MS/MS analysis. The samples were solubilized in 5 μL 30% formic acid and diluted with 40 μL eluent A (97.9% H_2_O, 2% acetonitrile, 0.1% formic acid). Samples analysis was carried out by nanoUHPLC–ESI-MS/MS applying a high resolution orbitrap mass spectrometer (Dionex Ultimate 3000 RSLCnano, Q Exactive orbitrap, Thermo Scientific). The data analysis was performed with Proteome Discoverer 1.3.0.339 by searching against the *J.* *regia* fasta file from the UniProt database (COLU17). The peptide mass tolerance was 5 ppm with a maximum number of 2 missed cleavages, carbamidomethylation of cysteines was set as static modification whereas oxidation of methionine was the only dynamic modification. For high confidence of the MS data, the false discovery rate (FDR) of the peptide spectrum matches (PSM) was set to <0.01 (Proteome Discoverer).

## Figures and Tables

**Table 1:** List of peptides found by nanoUHPLC–ESI-MS/MS protein identification experiments of *jrPPO1*(Asp^101^ → Pro^444^). Sequence coverage: 96%.

Start–End	Sequence	Modifications	XCorr	Delta Mass [ppm]	Enzyme for digestion	Peptide mass
101–108	DPVSAPEL		1.5	−0.93	Chymotrypsin	826.406
101–110	DPVSAPELTL		1.97	0.46	Chymotrypsin	1040.539
101–131	DPVSAPELTLcSEADLPAGALPVN**cc**PPTSK	C11(Carbamidomethyl); C25(Carbamidomethyl); C26(Carbamidomethyl)	6.51	−1.72	Trypsin	3265.524
111–136	**c**SEADLPAGALPVN**cc**PPTSKKIKDF	C1(Carbamidomethyl); C15(Carbamidomethyl); C16(Carbamidomethyl)	5.5	−1.18	Chymotrypsin	2874.368
122–136	PVN**cc**PPTSKKIKDF	C4(Carbamidomethyl); C5(Carbamidomethyl)	3.4	0.91	Chymotrypsin	1789.887
132–146	KIKDFVLPSQNTPLR		4.86	−2	Trypsin	1755.000
133–146	IKDFVLPSQNTPLR		5.05	−2.46	Trypsin	1626.905
135–146	DFVLPSQNTPLR		3.06	−118	Trypsin	1385728
139–153	PSQNTPLRVRPAAHL		1.62	2.05	Chymotrypsin	1655.925
146–153	RVRPAAHL		2.03	−1.21	Chymotrypsin	918.550
146–158	RVRPAAHLVDNDY		3	1.99	Chymotrypsin	1524.782
146–162	RVRPAAHLVDNDYIAKY		4.22	0	Chymotrypsin	2000.059
147–161	VRPAAHLVDNDYIAK		5.17	−1.54	Trypsin	1680.892
154–162	VDNDYIAKY		2.51	0.48	Chymotrypsin	1099.519
162–170	YNKGIEL**m**K	M8(Oxidation)	2.17	−1.16	Trypsin	1110.573
163–168	NKGIEL		1.85	−0.4	Chymotrypsin	672.380
163–180	NKGIEL**m**KSLPADDPRSF	M7(Oxidation)	5.07	−1.8	Chymotrypsin	2033.021
163–180	NKGIELMKSLPADDPRSF		4.73	−0.13	Chymotrypsin	2017.030
169–180	**m**KSLPADDPRSF	M1(Oxidation)	3.53	−0.13	Chymotrypsin	1378.654
169–180	MKSLPADDPRSF		2.91	−1.08	Chymotrypsin	1362.658
171–178	SLPADDPR		1.7	−1.54	Trypsin	869.422
173–180	PADDPRSF		1.65	−0.44	Chymotrypsin	903.408
181–190	TQQANVH**c**AY	C8(Carbamidomethyl)	2.21	−1.43	Chymotrypsin	1190.512
181–195	TQQANVH**c**AY**c**DGAY	C8(Carbamidomethyl); C11(Carbamidomethyl)	2.5	−1.49	Chymotrypsin	1756.690
196–203	TQVGFPDL		2.03	0.15	Chymotrypsin	875.439
196–205	TQVGFPDLSL		2.18	0.34	Chymotrypsin	1075.555
219–226	YYVYFFEK		2.61	−2.57	Trypsin	1157.540
219–230	YYVYFFEKILGK		2.12	−1.23	Trypsin	1568.825
224–231	FEKILGKL		2.68	0.42	Chymotrypsin	946.585
225–237	EKILGKLIGDPTF		3.38	−0.64	Chymotrypsin	1429.817
229–237	GKLIGDPTF		2.45	−0.14	Chymotrypsin	946.512
231–268	LIGDPTFALPFWNWDSPPG**m**QLPSLYAVSNSAIYDPLR	M20(Oxidation)	6.57	0.27	Trypsin	4264.099
231–268	LIGDPTFALPFWNWDSPPGMQLPSLYAVSNSAIYDPLR		4.55	−1.96	Trypsin	4248.094
245–256	DSPPG**m**QLPSLY	M6(Oxidation)	2.26	1.09	Chymotrypsin	1319.608
245–256	DSPPGMQLPSLY		2.72	0.57	Chymotrypsin	1303.612
257–267	AVSNSAIYDPL		1.69	−0.12	Chymotrypsin	1148.571
257–280	AVSNSAIYDPLRNANHQPPTIIDL		2.94	1.12	Chymotrypsin	2618.348
265–280	DPLRNANHQPPTIIDL		3.09	−1.55	Chymotrypsin	1812.945
268–280	RNANHQPPTIIDL		2.32	0.19	Chymotrypsin	1487.784
268–299	RNANHQPPTIIDLDYGETSESTTTTDQVPSNL		7.47	−3.7	Chymotrypsin	3513.636
269–300	NANHQPPTIIDLDYGETSESTTTTDQVPSNLK		7.62	−0.96	Trypsin	3485.640
281–299	DYGETSESTTTTDQVPSNL		2.95	2.68	Chymotrypsin	2043.881
300–316	KI**m**YRQ**m**VSGAKNPTLF	M3(Oxidation); M7(Oxidation)	1.47	0.1	Chymotrypsin	2015.033
301–311	I**m**YRQ**m**VSGAK	M2(Oxidation); M6(Oxidation)	2.21	−0.22	Trypsin	1314.642
304–316	RQ**m**VSGAKNPTLF	M3(Oxidation)	4.59	1.53	Chymotrypsin	1463.757
304–316	RQMVSGAKNPTLF		4.15	−0.15	Chymotrypsin	1447.760
304–317	RQ**m**VSGAKNPTLFF	M3(Oxidation)	3.57	0.8	Chymotrypsin	1610.825
304–317	RQMVSGAKNPTLFF		3.47	0.88	Chymotrypsin	1594.830
312–322	NPTLFFGSPYR		3.89	−2.13	Trypsin	1297.642
322–344	RAGDEPDPGAGTIESTPHNNIHL		6.32	−0.98	Chymotrypsin	2397.128
322–345	RAGDEPDPGAGTIESTPHNNIHLW		8.24	−0.92	Chymotrypsin	2583.207
345–361	WTGDDTQPNIEN**m**GNFY	M13(Oxidation)	1.9	2.95	Chymotrypsin	2016.821
346–360	TGDDTQPNIEN**m**GNF	M12(Oxidation)	3.18	0.09	Chymotrypsin	1667.673
346–360	TGDDTQPNIENMGNF		2.79	0.34	Chymotrypsin	1651.679
346–361	TGDDTQPNIENmGNFY	M12(Oxidation)	3.34	−0.1	Chymotrypsin	1830.736
346–361	TGDDTQPNIENMGNFY		3.3	2.27	Chymotrypsin	1814.745
361–370	YSAGRDPIFF		2.84	1.15	Chymotrypsin	1171.567
362–370	SAGRDPIFF		2.25	0.63	Chymotrypsin	1008.503
362–380	SAGRDPIFFAHHSNVDR**m**W	M18(Oxidation)	4.74	0.12	Chymotrypsin	2258.044
366–378	DPIFFAHHSNVDR		4.07	−2.16	Trypsin	1553.734
366–389	DPIFFAHHSNVDR**m**WTIWKTLGGK	M14(Oxidation)	3.01	−1.14	Trypsin	2871.424
371–380	AHHSNVDR**m**W	M9(Oxidation)	2.93	−1.01	Chymotrypsin	1267.550
371–380	AHHSNVDRMW		3.42	−0.36	Chymotrypsin	1251.556
371–383	AHHSNVDR**m**WTIW	M9(Oxidation)	4	−1.68	Chymotrypsin	1667.759
371–383	AHHSNVDRMWTIW		2.33	0.11	Chymotrypsin	1651.767
371–386	AHHSNVDR**m**WTIWKTL	M9(Oxidation)	2.48	−1.23	Chymotrypsin	2009.986
379–384	**m**WTIWK	M1(Oxidation)	1.88	−2.53	Trypsin	879.429
379–384	MWTIWK		1.88	−2.18	Trypsin	863.434
379–389	**m**WTIWKTLGGK	M1(Oxidation)	2.36	−1.39	Trypsin	1335.699
384–399	KTLGGKRKDITDPDWL		3.77	−1.36	Chymotrypsin	1841.997
387–399	GGKRKDITDPDWL		3.74	−0.94	Chymotrypsin	1499.771
387–403	GGKRKDITDPDWLNSSF		3.6	−0.87	Chymotrypsin	1934.946
390–414	RKDITDPDWLNSSFFFYDENADPVR		4.9	−0.72	Trypsin	3046.407
391–414	KDITDPDWLNSSFFFYDENADPVR		7.02	−1.3	Trypsin	2890.304
392–414	DITDPDWLNSSFFFYDENADPVR		6.48	−1.21	Trypsin	2762.210
392–416	DITDPDWLNSSFFFYDENADPVRVK		2.67	−0.2	Trypsin	2989.376
405–426	FYDENADPVRVKVKD**c**VDNTKL	C16(Carbamidomethyl)	6.06	2.94	Chymotrypsin	2624.298
406–426	YDENADPVRVKVKD**c**VDNTKL	C15(Carbamidomethyl)	7.26	−2.45	Chymotrypsin	2477.216
406–428	YDENADPVRVKVKD**c**VDNTKLRY	C15(Carbamidomethyl)	7.6	−1.21	Chymotrypsin	2796.383
407–426	DENADPVRVKVKD**c**VDNTKL	C14(Carbamidomethyl)	6.25	−1.18	Chymotrypsin	2314.156
407–428	DENADPVRVKVKD**c**VDNTKLRY	C14(Carbamidomethyl)	6.73	−0.72	Chymotrypsin	2633.321
415–425	VKVKD**c**VDNTK	C6(Carbamidomethyl)	1.48	−0.38	Trypsin	1304.675
417–427	VKD**c**VDNTKLR	C4(Carbamidomethyl)	3.09	0.1	Trypsin	1346.697
427–437	RYVYQDVEIPW		2.86	−0.83	Chymotrypsin	1466.718
428–439	YVYQDVEIPWLK		4.01	−0.47	Trypsin	1551.796
428–444	YVYQDVEIPWLKTKPTP		3.39	−0.83	Trypsin	2076.091
429–437	VYQDVEIPW		1.91	0.01	Chymotrypsin	1147.554
429–438	VYQDVEIPWL		1.72	−0.64	Chymotrypsin	1260.638
431–437	QDVEIPW		1.95	−0.85	Chymotrypsin	885.422
431–444	QDVEIPWLKTKPTP		3.47	−0.39	Chymotrypsin	1650.897
438–444	LKTKPTP		1.84	−0.35	Chymotrypsin	783.485

**Table 2:** List of peptides found by nanoUHPLC–ESI-MS/MS protein identification experiments of *jrPPO1*(Asp^101^ → Arg^445^). Sequence coverage: 96%.

Start–End	Sequence	Modifications	XCorr	Delta Mass [ppm]	Enzyme for digestion	Peptide mass
101–110	DPVSAPELTL		2.12	−0.71	Chymotrypsin	1040.5378
101–131	DPVSAPELTL**c**SEADLPAGALPVN**cc**PPTSK	C11(Carbamidomethyl); C25(Carbamidomethyl); C26(Carbamidomethyl)	6.56	−2.17	Trypsin	3265.5230
111–136	**c**SEADLPAGALPVN**cc**PPTSKKIKDF	C1(Carbamidomethyl); C15(Carbamidomethyl); C16(Carbamidomethyl)	5.93	2.45	Chymotrypsin	2874.3781
122–136	PVN**cc**PPTSKKIKDF	C4(Carbamidomethyl); C5(Carbamidomethyl)	2.56	0.8	Chymotrypsin	1789.8865
132–146	KIKDFVLPSQNTPLR		3.15	−1.79	Trypsin	1755.0007
133–146	IKDFVLPSQNTPLR		5.19	−2.01	Trypsin	1626.9056
135–146	DFVLPSQNTPLR		3.16	−0.74	Trypsin	1385.7288
146–153	RVRPAAHL		1.8	−0.32	Chymotrypsin	918.5504
146–158	RVRPAAHLVDNDY		3.16	0.21	Chymotrypsin	1524.7795
146–162	RVRPAAHLVDNDYIAKY		4.25	1.41	Chymotrypsin	2000.0615
147–161	VRPAAHLVDNDYIAK		5.28	−1.87	Trypsin	1680.8911
154–162	VDNDYIAKY		2.58	0.48	Chymotrypsin	1099.5186
163–180	NKGIEL**m**KSLPADDPRSF	M7(Oxidation)	4.69	−2.97	Chymotrypsin	2033.0187
169–180	**m**KSLPADDPRSF	M1(Oxidation)	3.27	−1.82	Chymotrypsin	1378.6521
169–180	MKSLPADDPRSF		2.88	−0.9	Chymotrypsin	1362.6585
171–178	SLPADDPR		1.84	−3.57	Trypsin	869.4207
181–190	TQQANVH**c**AY	C8(Carbamidomethyl)	1.95	−0.09	Chymotrypsin	1190.5132
181–195	TQQANVH**c**AY**c**DGAY	C8(Carbamidomethyl); C11(Carbamidomethyl)	2.55	−1.21	Chymotrypsin	1756.6907
196–205	TQVGFPDLSL		2.23	−0.68	Chymotrypsin	1075.5537
219–226	YYVYFFEK		2.67	−3.41	Trypsin	1157.5389
224–231	FEKILGKL		2.9	0.55	Chymotrypsin	946.5852
225–237	EKILGKLIGDPTF		1.9	0.48	Chymotrypsin	1429.8183
229–237	GKLIGDPTF		2.38	1.92	Chymotrypsin	946.5137
231–268	LIGDPTFALPFWNWDSPPG**m**QLPSLYAVSNSAIYDPLR	M20(Oxidation)	7.3	−2.7	Trypsin	4264.0861
231–268	LIGDPTFALPFWNWDSPPGMQLPSLYAVSNSAIYDPLR		4.64	0	Trypsin	4248.1027
245–256	DSPPG**m**QLPSLY	M6(Oxidation)	2.67	−1.87	Chymotrypsin	1319.6038
245–256	DSPPGMQLPSLY		2.09	0.48	Chymotrypsin	1303.6120
257–267	AVSNSAIYDPL		1.87	0.2	Chymotrypsin	1148.5711
257–280	AVSNSAIYDPLRNANHQPPTIIDL		2.56	−2.31	Chymotrypsin	2618.3388
265–280	DPLRNANHQPPTIIDL		2.99	−0.64	Chymotrypsin	1812.9466
268–280	RNANHQPPTIIDL		2.38	−1.54	Chymotrypsin	1487.7817
268–299	RNANHQPPTIIDLDYGETSESTTTTDQVPSNL		6.4	−1.69	Chymotrypsin	3513.6432
269–300	NANHQPPTIIDLDYGETSESTTTTDQVPSNLK		7.62	−0.5	Trypsin	3485.6412
281–299	DYGETSESTTTTDQVPSNL		2.5	0.95	Chymotrypsin	2043.8771
301–311	I**m**YRQ**m**VSGAK	M2(Oxidation); M6(Oxidation)	2.23	0.26	Trypsin	1314.6423
304–316	RQ**m**VSGAKNPTLF	M3(Oxidation)	4.59	2.34	Chymotrypsin	1463.7584
304–316	RQMVSGAKNPTLF		4.25	−3.56	Chymotrypsin	1447.7549
304–317	RQ**m**VSGAKNPTLFF	M3(Oxidation)	3.91	0.35	Chymotrypsin	1610.8240
304–317	RQMVSGAKNPTLFF		2.97	0.3	Chymotrypsin	1594.8290
312–322	NPTLFFGSPYR		3.87	−1.66	Trypsin	1297.6429
322–344	RAGDEPDPGAGTIESTPHNNIHL		7.11	−0.59	Chymotrypsin	2397.1290
322–345	RAGDEPDPGAGTIESTPHNNIHLW		8.28	−3.12	Chymotrypsin	2583.2017
346–360	TGDDTQPNIEN**m**GNF	M12(Oxidation)	3.01	−1.67	Chymotrypsin	1667.6701
346–360	TGDDTQPNIENMGNF		2.75	−2.03	Chymotrypsin	1651.6746
346–361	TGDDTQPNIEN**m**GNFY	M12(Oxidation)	3.17	−1.57	Chymotrypsin	1830.7333
346–361	TGDDTQPNIENMGNFY		3.05	−0.21	Chymotrypsin	1814.7409
361–370	YSAGRDPIFF		2.72	0.52	Chymotrypsin	1171.5663
362–370	SAGRDPIFF		2.18	−1.06	Chymotrypsin	1008.5013
362–380	SAGRDPIFFAHHSNVDR**m**W	M18(Oxidation)	4.16	−0.72	Chymotrypsin	2258.0419
366–378	DPIFFAHHSNVDR		4.06	−2.08	Trypsin	1553.7338
371–380	AHHSNVDR**m**W	M9(Oxidation)	3.04	−0.51	Chymotrypsin	1267.5505
371–383	AHHSNVDR**m**WTIW	M9(Oxidation)	2.42	0.27	Chymotrypsin	1667.7627
379–384	**m**WTIWK	M1(Oxidation)	1.92	−2.6	Trypsin	879.4285
379–384	MWTIWK		1.78	−1.26	Trypsin	863.4348
384–399	KTLGGKRKDITDPDWL		3.14	−0.9	Chymotrypsin	1841.9978
387–399	GGKRKDITDPDWL		3.04	−0.61	Chymotrypsin	1499.7719
387–403	GGKRKDITDPDWLNSSF		3.94	0.36	Chymotrypsin	1934.9489
391–414	KDITDPDWLNSSFFFYDENADPVR		7.56	−1.24	Trypsin	2890.3046
392–414	DITDPDWLNSSFFFYDENADPVR		7.01	−1.48	Trypsin	2762.2091
405–426	FYDENADPVRVKVKD**c**VDNTKL	C16(Carbamidomethyl)	6.25	−0.33	Chymotrypsin	2624.2891
406–426	YDENADPVRVKVKD**c**VDNTKL	C15(Carbamidomethyl)	7.3	−0.6	Chymotrypsin	2477.2201
406–428	YDENADPVRVKVKD**c**VDNTKLRY	C15(Carbamidomethyl)	5.94	−0.41	Chymotrypsin	2796.3849
407–426	DENADPVRVKVKD**c**VDNTKL	C14(Carbamidomethyl)	6.03	−0.71	Chymotrypsin	2314.1566
407–428	DENADPVRVKVKD**c**VDNTKLRY	C14(Carbamidomethyl)	6.48	2.13	Chymotrypsin	2633.3283
417–427	VKD**c**VDNTKLR	C4(Carbamidomethyl)	2.81	−0.1	Trypsin	1346.6970
427–437	RYVYQDVEIPW		2.69	−1	Chymotrypsin	1466.7174
428–439	YVYQDVEIPWLK		4	−2	Trypsin	1551.7937
429–437	VYQDVEIPW		1.98	−0.63	Chymotrypsin	1147.5537
431–437	QDVEIPW		1.71	−0.37	Chymotrypsin	885.4224
431–445	QDVEIPWLKTKPTPR		3.9	−0.39	Chymotrypsin	1806.9981
438–445	LKTKPTPR		1.88	0.23	Chymotrypsin	939.5863

**Table 3:** Kinetic parameters for the monophenolase and diphenolase activity of *Juglans regia* tyrosinase *jrPPO1*(Asp^101^ → Arg^445^).

Substrate	*λ* [nm]	*ε* [M^−1^ cm^−1^]	*K*_m_ [mM]	*V*_m_ [mM min^−1^]	*V*_m_/*K*_m_ [min^−1^]	*k*_cat_ [s^−1^]	*k*_cat_/*K*_m_ [mM^−1^ s^−1^]
l-tyrosine	475	3600⁎	1.9	0.08	0.04	20.8	10.9
l-dopa	475	3600⁎	8.8	0.06	0.01	199.3	22.8

⁎ Values taken from Gandía-Herrero et al. (2005).
